# Coping Strategies in Male Patients under Treatment for Substance Use Disorders and/or Severe Mental Illness: Influence in Clinical Course at One-Year Follow-Up

**DOI:** 10.3390/jcm8111972

**Published:** 2019-11-14

**Authors:** Julia E. Marquez-Arrico, Laura Río-Martínez, José Francisco Navarro, Gemma Prat, Diego A. Forero, Ana Adan

**Affiliations:** 1 Department of Clinical Psychology and Psychobiology, School of Psychology, University of Barcelona, Passeig de la Vall d’Hebrón 171, 08035 Barcelona, Spain; jmarquez@ub.edu (J.E.M.-A.); laurario@ub.edu (L.R.-M.); gprat@ub.edu (G.P.); 2 Institute of Neurosciences, University of Barcelona, 08035 Barcelona, Spain; 3 Department of Psychobiology, School of Psychology, University of Málaga, Campus de Teatinos s/n, 29071 Málaga, Spain; navahuma@uma.es; 4 Laboratory of NeuroPsychiatric Genetics, Biomedical Sciences Research Group, School of Medicine, Universidad Antonio Nariño, Bogotá 110231, Colombia; diego.forero@uan.edu.co; 5 School of Health Sciences, Fundación Universitaria del Área Andina, Bogotá 110231, Colombia

**Keywords:** coping, substance use disorder, dual diagnosis, relapses, schizophrenia, major depressive disorder

## Abstract

Coping strategies have an impact on substance use disorders (SUD), relapses, and clinical variables, but knowledge on this area is scarce. We explored the coping strategies used during treatment in patients with dual diagnosis (DD), SUD, and severe mental illness (SMI), and the relation with clinical course and relapses at one-year follow-up. A sample of 223 patients was divided into three groups depending on diagnosis: DD (*N* = 80; SUD with comorbid schizophrenia or major depressive disorder), SUD only (*N* = 80), and SMI only (*N* = 63; schizophrenia or major depressive disorder). MANCOVA analyses reflected differences in self-criticism and problem avoidance, with a higher use of these in the DD and SUD groups. The coping strategies used differed depending on the presence/absence of a SUD, but not depending on psychiatric diagnosis. At one-year follow-up, social support was the only strategy that predicted the presence of relapses in DD patients with schizophrenia (positively), and in SMI patients with major depressive disorder (negatively). Thus, social support was associated with relapses, but the relationship was different depending on psychiatric diagnosis. Further studies should analyze the implications of social support as a coping strategy in different mental disorders, as well as its usefulness in individualized interventions.

## 1. Introduction

During recent years, scientific interest in how people cope with stress and adversity has increased significantly, since coping strategies are known to have an influence on the differences observed among people under stress, and have proven their importance as a stabilizing factor, facilitating individual adjustment and leading to better health [1,2,3]. 

Coping strategies are understood as specific actions that individuals use in order to manage, change, and/or reduce the consequences of exposition to stressors [4,5]. It is widely accepted that task-oriented coping strategies are associated with less complications, while emotion-oriented strategies are linked to the risk of experiencing anxiety and depressive symptoms, health problems, and drug use [6,7]. 

One of the main areas of study regarding coping strategies is their relationship with substance use disorders (SUD), since the use of adaptive strategies during stressful situations or events is a key factor in how individuals respond and their possible substance use [8]. When confronted with new stressors, people with limited coping strategies have a higher probability of using drugs than those with a wider coping repertory, because the former tend to use psychoactive drugs in order to cope with negative emotions and stress [9]. Among people who use drugs, a very high percentage use the substance itself as self-medication to confront daily stressful situations [10]. In general, this avoidance coping pattern is a strategy positively associated with drug use [11,12], whereas engaging in adaptive coping is related to a decrease in substance abuse, improving SUD treatment effects [11]. In this sense, the use of dysfunctional strategies in patients with SUD has been linked to the severity of their addiction [13,14] and the frequency of relapses [13,15]. 

Furthermore, numerous studies have confirmed the high comorbidity between SUD and severe mental illness (SMI). This condition, commonly known as dual diagnosis (DD), is especially observed among patients with major depressive disorder and schizophrenia spectrum disorders. In this context, up to 50% of patients with such psychopathologies are also diagnosed with a comorbid SUD [16,17]. DD is more common in males [18,19], is associated with major clinical and social complications [20,21], worse treatment adherence [22,23], higher suicide rates [24,25,26], and more relapses [26,27,28], when compared to single-diagnosis psychiatric conditions. Hence, DD is a complicated clinical condition for both patients and mental health professionals.

Despite the strong relationship between SUD and coping, and the clinical implications on the strategies used by patients to cope with stressful events, very few studies have explored coping strategies in DD patients, and even fewer have considered the type of SMI diagnosed. Available studies show how DD patients with schizophrenia or major depression disorder tend to use more maladaptive coping when compared to patients with only a SUD diagnosis [27,29,30]. While SUD with comorbid schizophrenia is associated with a lower use of problem solving, self-criticism, and social support strategies [27], SUD with comorbid major depression disorder is linked to a higher use of social withdrawal and a lower use of cognitive restructuring strategies [31]. In general, schizophrenia and major depressive disorder patients use more avoidance and reality-escaping strategies, show more dependence from others, excessive expression of emotions, and poorer thinking processes [32,33,34]. 

Moreover, in both DD and SMI patients with major depressive disorder or schizophrenia, training and encouraging the use of coping strategies oriented to problem solving and emotional regulation is associated with a decrease in drug abuse and suicidal attempts [33,35], improving their management of stress and perception self-efficacy [33,36]. More specifically, previous works have confirmed that learning adaptive strategies during SUD treatment has a stronger effect on reducing relapses and decreasing clinical symptoms associated with the comorbid SMI [37,38,39].

Considering the insufficient knowledge on the use of coping strategies in patients with DD, we decided to examine whether there were any differences in the use of such strategies among our SUD, DD, and SMI groups, given that DD and SMI are the two most prevalent psychiatric diagnoses in SUD patients. We then compared the results with the Spanish normative data, to check whether our groups were within the norm. Additionally, we explored if any of the strategies considered had a predictive value on patients’ relapses within a one-year follow-up, since relapses are one of the main problems in the recovery of DD patients [24,28,40,41]. To our knowledge, this is the first study to establish comparisons in the use of coping strategies in these three groups of patients (DD, SMI, SUD), in contrast with other studies that have done so in groups of only-SUD or only-SMI patients. Moreover, it is the first one to investigate such strategies as possible predictors of relapse in these groups at one-year follow-up.

## 2. Experimental Section

### 2.1. Participants

The total sample consisted of 223 voluntary male patients, assigned to three groups according to their clinical diagnoses: DD group (*N* = 80), SUD (*N* = 80), and SMI (*N* = 63). The DD group had 37 patients with schizophrenia (46.3%) and 43 with major depressive disorder (53.7%), and the SMI group had 32 patients with schizophrenia (50.8%) and 31 with major depressive disorder (49.2%). Our study is a multicenter research, with participants from different public and private clinical centers specialized in SMI and SUD treatments. All patients were in therapeutic programs for their corresponding diagnoses and had obtained negative results in all their abstinence urine analyses. 

Our inclusion criteria were: (1) Male gender (based on the greater prevalence of men in SUD and DD diagnoses, and also due to their larger presence in our clinical centers); (2) aged 19 to 55 years; (3) currently under treatment for their mental disorders, but in a clinically stable condition; (4) the diagnoses of major depressive disorder or schizophrenia in the DD and SMI groups had been established according to DSM-5 criteria [42]; (5) a current diagnosis of SUD for the DD and SUD patients, including those with addiction in early remission according to DSM-5 criteria (abstinence period from 3 to 12 months, without relapses in the last 3 months) [42]. The exclusion criteria were: (1) Meeting DSM-5 criteria for a current substance-induced disorder; (2) psychiatric condition due to medical disease; (3) unstable or uncontrolled symptomatology; (4) inability to complete study interviews or instruments.

### 2.2. Procedure 

Patients were referred to our study from their respective treatment centers by their treating psychiatrists or psychologists, who followed our inclusion/exclusion criteria for referral. Once the participants agreed to be subjects in our study, they were provided and signed an informed consent. Then, a postgraduate psychologist from our research group conducted the assessment protocol of our study in two sessions. The participants were not compensated for their participation and the only benefit they obtained was a report of their results. The assessment protocol was approved by the Research Committee of the University of Barcelona (IRB00003099) and is part of a wider research project. The present study complied with the tenets of the Declaration of Helsinki.

For the follow-up, the same postgraduate psychologist visited each treatment center at 3, 6, and 12 months to collect the questionnaires completed by each patient in the sample. The follow-up data were collected using a structured 21-item questionnaire, specifically designed for our study. The main variables registered were relapses (presence/absence), patients’ treatment status, suicide attempts (yes/no), and number of medical consultations (outside the patients’ main treatment facilities) during the follow-up period. 

### 2.3. Measures

#### 2.3.1. Sociodemographic and Clinical Variables

A structured interview specifically designed for the present study was conducted with each patient to assess variables such as age, civil status, years of schooling, social situation, and living arrangements, among others. This interview was also employed to collect data on diagnosis according to DSM-5 criteria, personal/family psychiatric and medical records, history of suicide attempts, and SMI/SUD age of onset. In addition, the structured clinical interview for DSM-IV-TR Axis I Disorders (SCID-I) [43] was administered to collect other clinical variables such as medication prescribed, hospitalizations, type and number of drugs used, and abstinence period. The SCID-I for DSM-IV-TR was used since the corresponding Spanish version for the DSM-5 was not available at the time. 

The drug abuse screening test (DAST-20) [44] was used to obtain a measure of the SUD characteristics in the DD and SUD groups. The DAST-20 provides a total severity score ranging from 0 to 20 (1–5 low; 6–10 intermediate; 11–15 substantial; 16–20 severe), with higher scores indicating that a more intensive therapeutic intervention was recommended.

Regarding psychiatric symptoms, the schizophrenia patients in the DD and SMI groups were administered the positive and negative syndrome scale (PANSS, Spanish version) [45]. The PANSS scale yields scores in four areas related to different symptomatology: Positive syndrome, negative syndrome, composite scale, and general psychopathology. Depressive symptoms in patients with major depressive disorder in the DD and SMI groups were measured with the Spanish version of the 17-item Hamilton depression rating scale (HDRS) [46], its cut-off points being: 0–7, no current depression; 8–13, low; 14–18, mild; 19–22, severe; and >23, very severe depressive symptoms [47]. 

#### 2.3.2. Coping Strategies Assessment

Coping strategies were assessed through the coping strategies inventory (CSI) [48] in its Spanish version [49], since it offers a valid and reliable measure of the strategies used in different stressful situations. It consists of 40 self-reported Likert items assessing different engagement and disengagement coping strategies. The CSI has primary, secondary, and tertiary scales, obtained by factor analysis. The eight primary scales are: Problem solving (strategies used to eliminate or change the stressful situation); cognitive restructuring (strategies used to change the subject’s interpretation of the stressful event); social support (coping efforts to obtain support from other people); express emotions (externalizing emotional experiences to cope with the stressful event); problem avoidance (coping efforts including denial and avoidance thoughts or behaviors related to the problem or its solution); wishful thinking (cognitive strategies reflecting a desire for reality to be different); social withdrawal (behaviors of isolation from family and friends or strategies for being alone when facing a stressful process); and self-criticism (self-blaming and guilt regarding the problem).

The four secondary scales are: Problem engagement, emotion engagement, problem disengagement, and emotion disengagement. The two tertiary scales are engagement and disengagement Whereas in the Spanish version of the CSI, the scores from the primary scales can be transformed into percentiles, the secondary and tertiary scales did not show strong psychometric properties, and there are no normative data for them [49], so the latter scales were not considered in our analyses. 

### 2.4. Data Analysis

Descriptive statistics (frequencies, means, and standard errors) were obtained for the sociodemographic and clinical variables, and differences in such variables among groups were explored by univariate analyses of variance (ANOVA) for continuous data. Nonparametric tests were conducted, including Chi-Square, Kruskal–Wallis, and Mann–Whitney tests, depending on the type of variable analyzed. Multivariate analyses of covariance (MANCOVA) were performed introducing the primary scales of the CSI as dependent variables, and the group (SUD, DD, and SMI) as the independent variable, in order to detect differences in the strategies used by the three groups. Post-hoc comparisons were adjusted by Bonferroni’s correction. Other MANCOVA analyses were carried out to compare the results in each group according to their psychiatric diagnoses (DD and SMI), and to elucidate the role of schizophrenia and major depressive disorder in them. In all the variance analyses, age was considered as a covariate to control its possible effect, given that our groups differed significantly in this variable, the SMI patients being older on average. The coping strategies used in each group were also compared, using the Spanish normative data available for the primary scales of the CSI [49]. 

We also explored the differences in coping regarding addiction relapses during the 12-month follow-up period with MANCOVA analyses. The independent variables were diagnosis group and presence/absence of relapses at one-year follow-up, the dependent variables were the primary coping scales, and age was a covariate. The possible inter-group differences for coping strategies in the DD and SMI groups were also explored, according to psychiatric diagnosis and relapses at one-year follow-up. In all the MANCOVA analyses, the partial eta square *η_p_*^2^ was obtained as a measure of the effect size, the cut-off points being 0.01 (small), 0.04 (medium), and 0.1 (large) [50]. 

Finally, logistic regression models were applied to determine possible predictors of the results in each group for the one-year follow-up. Logistic regression coefficients and their standard errors were back-transformed to generate odds-ratios (ORs) and their 95% confidence intervals. All statistical analyses were carried out using the SPSS/PC+ statistics package (version 17.0, SPSS Inc., Chicago, United States), and tests were two-tailed with the type I error set at 5%. 

## 3. Results

### 3.1. Sociodemographic and Clinical Characteristics

Sociodemographic data for the three groups are shown in Table 1. Mean age of the total sample was 39.11 ± 8.45 years old. Most of the subjects had completed primary studies, were single, and currently living with their family. We observed differences among the groups in age (*p* = 0.004), civil status (*p* = 0.008), living arrangements (*p* = 0.006), and employment situation (*p* = 0.015). The SMI group had an older mean age, and more SMI patients were living with friends or in protected flats. Having a disability pension was associated with DD and SMI diagnoses (>60%), while being employed was associated with a single diagnosis of SUD. 

Regarding clinical data (see Table 2), there were differences in the onset age of SUD, with the DD group having an earlier onset of addiction (*p* = 0.023), and also in the onset age of SMI, with the DD patients being younger at first diagnosis of SMI, on average (*p* = 0.030). However, the duration of the SUD and the SMI did not show significant differences between the two groups. In addition, the SUD patients were less likely to be using psychiatric medication (*p* = 0.007) and had a lower medical disease comorbidity rate (*p* = 0.022) than the DD and SMI patients. 

The DD and SUD groups showed no differences in severity of addiction regarding the scores in the DAST-20 or the months of abstinence. The most prevalent substances of abuse were cocaine and alcohol, with a high rate of polydrug use (up to 56%) in both groups. However, there were differences in the composite scale of PANSS for those patients with schizophrenia, depending on whether they were in the DD or the SMI group. In this sense, the SMI patients showed a higher predominance of negative symptoms than the DD patients (*p* = 0.040). Finally, no differences in the HDRS results were observed for those patients with major depressive disorder. 

### 3.2. Coping Strategies in the DD, SUD, and SMI groups

CSI scale scores in the DD, SUD, and SMI groups, and the comparisons among them, are shown in Table 3. The MANCOVA analyses indicate significant differences among groups in the scales of self-criticism and problem avoidance. The DD and SUD groups obtained higher scores in self-criticism (*p* < 0.001), and lower scores in problem avoidance (*p* < 0.001), when compared to the SMI group. In contrast, no differences in the coping scales were found between the DD and SMI groups regarding psychiatric diagnosis (schizophrenia or major depressive disorder), or regarding the presence or absence of SUD (*p* ≥ 0.281 in all cases).

**Figure 1:**
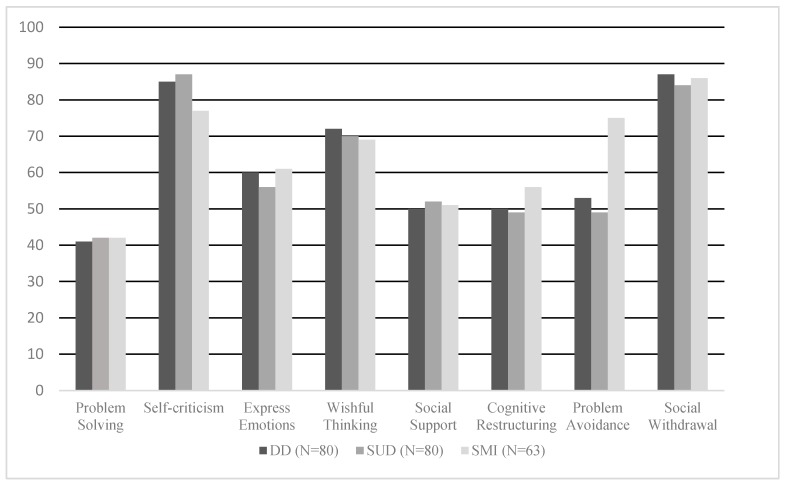
Percentile scores for the three groups in each of the primary scales of the coping strategies inventory (CSI), according to Spanish normative data. DD: Dual diagnosis; SUD: Substance use disorder; SMI: Severe mental illness.

When considering the Spanish normative data, an analysis of percentiles (see Figure 1) showed that the three groups presented a more frequent use (percentiles 70 to 80) of self-criticism, wishful thinking, and social withdrawal, and a less frequent use (percentile 40) of problem solving. In addition, when compared to the Spanish normative data, the SMI group presented a predominant use (percentile 75) of problem avoidance, whereas the scores in the DD and SUD groups were similar to norms for this strategy.

### 3.3. Follow-Up Data at Three, Six, and Twelve Months

Comparisons among the three groups detected differences regarding addiction relapses at 3, 6, and 12 months (see Table 4), their presence being higher in the DD and SUD groups, with a rate of approximately 50% at one-year follow-up. The highest relapse rate in the DD group was found in those patients with major depressive disorder. The SMI group had the lowest relapse rates in each follow-up, “relapse” being understood here as a new episode of the corresponding mental disorder (schizophrenia or major depressive disorder). 

Regarding the patients’ treatment status, we did not observe any differences at three months follow-up. However, the DD and SMI patients were more likely to be still in treatment than those with SUD at 6 (*p* = 0.035) and 12 months (*p* = 0.008), with the SMI patients also being more likely to be discharged from treatment. In addition, suicide attempts did not differ among the groups at three months, but they showed differences at 6 (*p* = 0.040) and 12 months (*p* = 0.007). At six months follow-up, a higher rate of suicide attempts was observed in the DD and SUD groups. SUD and SMI patients showed similar rates, although the highest was observed in the DD group at 2 months, especially for those patients with major depressive disorder. Overall, 21.8% of the DD patients had one suicide attempt at one-year follow-up. 

Finally, in all the follow-up periods, the DD group presented the highest number of medical consultations outside their main treatment facilities (*p* < 0.001), the SUD group showed the lowest quantity (*p* < 0.001, in all cases), with the SMI group being in the middle position.

### 3.4. Differences in Coping Strategies Considering One-Year Follow-Up Data

MANCOVA analyses were performed to explore the possible differences in coping strategies in the DD, SUD, and SMI groups at one-year follow-up in relation to the presence/absence of relapses. The results revealed differences in social support (*F*_(2,221)_ = 3.258; *ηp^2^* = 0.033; *p* = 0.041), with a lower score in those DD patients who had suffered relapses. With respect to relapses, no other differences in coping were observed among the DD, SUD, and SMI groups at one-year follow-up. 

When the coping strategies in the DD group were further analyzed, considering relapses at one-year follow-up and psychiatric diagnosis, differences were found in self-criticism (*F*_(1,78)_ = 4.356; *ηp^2^* = 0.059; *p* = 0.041) and social withdrawal (*F*_(1,78)_ = 10.206; *ηp^2^* = 0.127; *p* = 0.002). The DD patients with schizophrenia who had suffered relapses (14.82 ± 1.91) obtained a higher score in self-criticism. In contrast, in the DD patients with major depressive disorder, a higher score in self-criticism was obtained by those who had not relapsed (14.75 ± 1.12). Furthermore, for social withdrawal, patients with schizophrenia who had relapsed (14.48 ± 1.38) obtained a higher score, whereas in the major depressive disorder patients, a higher score was obtained by those who did not relapse at one-year follow-up (12.10 ± 1.07).

The analysis of strategies in the SMI group, considering relapses at one-year follow-up and psychiatric diagnosis, yielded differences in social support (*F*_(1,61)_ = 12.079; *ηp^2^* = 0.175; *p* = 0.001), with a lower score for schizophrenia patients with relapses (8.85 ± 1.84), while in the major depressive disorder patients, the lower score in social support was obtained by those who had not relapsed (9.24 ± 0.99).

### 3.5. Coping Strategies as Predictors of Relapses at One-Year Follow-Up 

Using logistic regression analyses, we analyzed the role of all these strategies in relapses at one-year follow-up for the three groups of patients. The only significant result was found for the social support strategy in the DD and SMI groups. In contrast, no primary coping strategies were observed as a significant predictor of relapses in the SUD group at one-year follow-up. 

When exploring the predictive value of social support in relapses for the DD group, a negative association was found in the schizophrenia patients (*β* = −0.153; *OR* = 0.858; *CI* 95.0% = 0.753–0.978; *p* = 0.022). In contrast, the relationship between this strategy and relapses was not significant (*p* = 0.750) for the DD patients with major depressive disorder at one-year follow-up. Furthermore, in the SMI group, social support was positively associated with relapses in those patients with major depressive disorder (*β* = 0.373; *OR* = 1.452; *CI* 95.0% = 1.098–1.919; *p* = 0.009), whereas this strategy was not a significant predictor of relapses for those patients with schizophrenia in the SMI group (*p* = 0.613).

Regarding the other clinical variables, the coping strategies analyzed in our study did not function as significant predictors of the patients’ treatment situation (whether in treatment, drop-out, discharged, or dismissed), suicide attempts, or number of medical consultations at any follow-up period (*p* ≥ 0.096 in all regression analyses).

## 4. Discussion

Our study focused on the coping strategies used by patients with DD, SUD, and SMI, also comparing the strategies used by each group in reference to the Spanish normative data. Furthermore, we analyzed if there were any differences in coping depending on psychiatric diagnosis for the DD and SMI groups, aiming to identify if any strategy could be a predictor of relapses at one-year follow-up. To our knowledge, this is the first study to present follow-up data at 3, 6, and 12 months in DD, SUD, and SMI patients. 

We observed that the sociodemographic and clinical characteristics of the patients in our sample were similar to those found in previous studies [21,24,26]. Having DD or SMI was associated with being single, having a disability pension, a higher probability of medical disease comorbidities, and a greater daily use of psychiatric medication. Furthermore, patients in the DD group had an earlier onset of SUD and SMI, which could imply major complications, since previous studies have found a relationship between a young age of SUD/SMI onset, psychosocial problems [51,52], and worse clinical symptomology [12,53,54].

Regarding our findings for coping strategies, patients differed in self-criticism and problem avoidance, depending on the presence of an addictive disorder. Hence, patients in the DD and SUD groups showed a stronger tendency to use coping efforts characterized by self-blaming and guilt (self-criticism) when facing problems, in contrast with patients with SMI only. This observation is in line with previous findings [31] and could be related to those SMI patients with schizophrenia only, who are more likely to have insight difficulties [34]. Moreover, patients in our sample with DD and SUD used denial and avoidance of thoughts or behaviors related to the problem (problem avoidance) as a coping strategy, with a higher frequency than patients with SMI only. Therefore, these strategies used by patients with DD and SUD seem to confirm that substance use is linked to disengagement strategies. In this sense, our findings are in agreement with previous research that has strongly related avoidance strategies to substance use [8,11,12,27,31,35], with patients in our sample differing in coping depending on the presence of an addictive disorder, but not depending on their psychiatric diagnosis. 

The comparison with the Spanish normative data for the coping strategies pointed out that, independently from the diagnostic group, when coping with stress all patients tend to use self-criticism (self-blame and guilt), wishful thinking (cognitive strategies that reflect a desire for reality to be different), and social withdrawal (isolation from friends and/or family, being alone). Thus, patients with DD, SUD, and SMI may be using disengagement strategies that are associated with unsuccessful ways of coping [7,15,29], which could interfere negatively with treatment compliance and relapse prevention therapy. 

Furthermore, the analyses of our data at 3, 6, and 12-month follow-up provided interesting results in relation to possible differences among the DD, SUD, and SMI groups when taking into account the presence of SUD and/or SMI. Consistent with previous research [17,22,23], we observed that DD patients had the highest relapse rates at all follow-ups. When exploring the differences regarding psychiatric diagnosis in the DD and SMI groups at one-year follow-up, we observed the highest relapse rate in patients with major depressive disorder, especially in the DD group. Moreover, at one-year follow-up, the DD group had the highest rate of patients still in treatment, a significant amount of suicide attempts, and the highest number of medical consultations. The presence of more clinical complications in our sample of DD patients is, thus, consistent with previous data [28,38,40]. These results emphasize the need to integrate treatment approaches in specialized DD units, which target both SUD and SMI simultaneously and as a whole [40,55].

We also explored the possible influence of coping among the DD, SUD, and SMI groups at one-year follow-up, taking into account the presence of relapses. The results showed that the use of the social support strategy was lower in those DD patients who had suffered relapses; that is, these patients presented a less frequent use of seeking people’s support as a coping strategy. Thus, having two comorbid conditions like SUD and schizophrenia, or like SUD and major depressive disorder, seems to pose notable difficulties in asking for support, or in preserving a strong social network that would give such support when needed [32,33,34,36].

When we further analyzed the strategies used by DD patients, together with relapses and psychiatric diagnosis, we observed differences in their tendency to experience self-blame and guilt (self-criticism), and to isolate from others (social withdrawal). These two strategies seemed to play a different role depending on the psychiatric diagnosis of the DD patients, since those with schizophrenia who had suffered relapses showed a higher use of self-criticism and social withdrawal, whereas a higher use of these strategies was observed in those with major depression disorder but no relapses. Our results for DD patients with schizophrenia are in line with previous data [14,33,35], while the results for DD patients with major depression disorder are unexpected, since those who used more often the coping strategy of self-blaming and isolation from others did not suffer any relapses. A possible explanation for these findings might be that those patients with major depressive disorder comorbid with SUD find these coping tendencies helpful to achieve the remission phase. Future studies should explore the role of coping depending on psychiatric comorbidity in patients with SUD to identify different effects and to develop an explanatory hypothesis. Our results suggest that including coping strategies in relapse prevention programs in order to improve the results at the end of treatment [37,38,39] would benefit only those patients with a comorbid diagnosis of schizophrenia, but not those with a major depressive disorder. In this sense, programs enhancing awareness and responsibility (self-criticism) and the ability to keep away from environmental influences (social withdrawal) may help patients with DD and depression to prevent relapses.

For SMI patients, social support was the only strategy that presented differences when taking into account psychiatric diagnosis and relapses at one-year follow-up. SMI patients with schizophrenia who had suffered relapses showed a lesser use of asking for help as a coping strategy, while those with major depressive disorder who had not suffered any relapses also presented a lesser use of this strategy. Thus, this is the first work presenting data about social support as a coping strategy that might have a different impact on how patients deal with problems depending on whether they have schizophrenia or major depressive disorder. Our findings support the idea of working towards a precision model in psychiatry [56], which considers the heterogeneity that may be observed among patients with SUD and other psychiatric comorbidities, as well as with SMI like schizophrenia or major depressive disorder. In this sense, future studies analyzing candidate genes might identify novel mechanisms involved in the relationships between coping, drug use, and clinical presentation of major psychiatric disorders [57,58], and thus be relevant in the advance of this field of work.

When we explored the strategies and their predictive value for relapses at one-year follow-up, we found that social support was linked to relapses in patients with DD and SMI, but not in patients with SUD only. Social support seemed to have a protective value for those DD patients with schizophrenia, but not for those DD patients with major depression disorder. This result is not consistent with previous data pointing out that social support is related to a better mental health [2,3], and suggest that promoting the use of asking for help as a coping strategy may not be useful when working with patients with SUD only. Asking for help or sharing problems with other people was negatively associated with relapses and had a possible protective effect in DD patients with schizophrenia. In contrast, this possible effect was not observed when depression was the psychiatric diagnosis. On the other hand, social support in the SMI group was a predictor of relapses at one-year follow-up only for patients with major depression disorder; that is, asking for help in these patients was positively linked to relapses. Thus, future studies should explore whether social support as a coping strategy is experienced differently according to psychiatric diagnosis, since these data could provide information to clinical practitioners when developing therapeutic interventions. 

Our study has a few strengths and some potential limitations. One strong point is the fact that we present follow-up data up to one year for a large sample of patients with DD, SUD, and SMI, and we associate these data with the strategies used by these patients. Our results also point out that, although social support has been considered traditionally as a strategy to prevent relapses, its effect may not be positive in all DD or in SMI patients. As potential limitations, we measured coping using a self-reported questionnaire, only once and not during follow-up periods, so we cannot compare whether gaining months of abstinence or being under treatment changes the coping strategies used by each diagnostic group. In addition, only male patients were included in our sample, and the age range was considerable. Moreover, the fact that all the patients were included in our study under the condition of being clinically stable, in order to prevent their participation from interfering in their therapeutic process, limits the clinical applicability of our findings. Future studies should consider these limitations in order to advance our knowledge in this area, and to provide clinicians with relevant data to improve treatment in relapse prevention programs. 

## 5. Conclusions

To our knowledge, this is the first study that compares coping strategies in patients with DD, SUD, and SMI, including the two most prevalent comorbid disorders (schizophrenia and major depressive disorder). It also considers how coping may differ among these groups and how some strategies might influence the presence of relapses at one-year follow-up. More specifically, the strategies of self-criticism and problem avoidance were used differently among the three groups depending on the presence of an addictive disorder, but not depending on the psychiatric condition. Thus, the SUD and DD groups showed a higher use of these strategies than the SMI patients did. Regarding the treatment of addictions, some clinical implications of these findings are the need to tailor treatment programs to reduce such disengagement strategies, and to train patients in coping that leads to an active problem-solving approach. Thus, treatment for SUD could include sessions following the solution-focused therapy model or the problem-solving therapy model, which are compatible with the approaches most currently used in addiction treatment.

Overall, when referring to the follow-up data, the DD group presented the type of clinical complications (earlier SUD/SMI onset, relapses, suicide attempts, more medical consultations) previously associated with poorer prognosis and treatment outcomes. Moreover, taking into account the follow-up data for relapses at one year, we observed that only the strategy of social support had a predictive value. The protective effect of this strategy seems to be mediated by the presence/absence of SUD, as well as by the type of SMI (schizophrenia or major depressive disorder), with benefits only for those patients with SUD and comorbid schizophrenia. Regarding the other diagnoses studied here, treatment and follow-up programs in clinical practice may consider that promoting social support will not always be a therapeutic approach for patients to cope with stressors in the early remission phase, especially for those with major depressive disorder. Future studies should consider our findings to improve available knowledge in this area, in order to design more precise and effective therapeutic interventions and relapse prevention programs. 

## Figures and Tables

**Table 1:** Sociodemographic data for the three groups, with means, standard deviation or percentages, and statistical contrasts.

Sociodemographic Variables	DD (*N* = 80)	SUD (*N* = 80)	SMI (*N* = 63)	Statistical Analyses
Age	38.71 ± 0.91	37.41 ± 0.89	41.90 ± 1.51	*F*_(2,221)_ = 5.768 *
Civil status				χ^2^_(4)_ = 7.754 **
Single	71.25%	52.20%	75.40%	
Married/Stable partner	8.70%	20.30%	7.50%	
Divorced/Separated	20.05%	27.50%	17.10%	
Living arrangements				χ^2^_(4)_ = 11.643 **
Alone	22.60%	25.50%	8.10%	
With family/partner/parents	58.10%	47.30%	51.40%	
With friends/protected flats	19.40%	27.20%	40.50%	
Employment situation				χ^2^_(4)_ = 19.388 **
Working	17.40%	45.10%	22.10%	
Unemployed	21.30%	35.50%	12.90%	
Disability pension	61.30%	19.40%	65.00%	
Education				χ^2^_(4)_ = 3.286
Primary	56.60%	45.80%	51.20%	
Secondary	26.30%	34.70%	35.90%	
University	17.1%	19.50%	12.90%	

DD: Dual diagnosis; SUD: Substance use disorder; SMI: Severe mental illness; * *p* < 0.05; ** *p* < 0.01.

**Table 2:** Clinical data for the three groups, with means, standard deviation or percentages, and statistical contrasts.

Clinical Variables	DD (*N* = 80)	SUD (*N* = 80)	SMI (*N* = 63)	Statistical Analyses
Age of SUD onset	15.72 ± 2.51	17.38 ± 5.80		*F*_(2,158)_ = 6.294 *
Age of SMI onset	24.22 ± 9.05		28 ± 10.78	*F*_(2,121)_ = 5.976 *
Duration of SUD (years)	20.62 ± 8.11	22.16 ± 8.15		*F*_(2,158)_ = 1.317
Duration of SMI (years)	15.64 ± 6.34		13.45 ± 3.49	*F*_(2,121)_ = 1.745
Medical disease comorbidity ^a^	44%	25%	43%	χ^2^_(2)_ = 8.011 *
Respiratory	16%	15%	22%	
Metabolic	28%	16%	38%	
Infectious	12%	28%	8%	
Other	44%	41%	32%	
Main substance of dependence				χ^2^_(2)_ = 4.483
Alcohol	33.8%	25%		
Cannabis	18.8%	11.3%		
Cocaine	43.8%	61.3%		
Opioids	3.8%	2.5%		
Polydrug use	56%	50%		χ^2^_(2)_ = 2.115
Abstinence period (months)	7.48 ± 2.74	8.29 ± 2.70		*F*_(2,158)_ = 0.219
DAST-20 (severity of addiction)	13.04 ± 1.15	12.30 ± 2.46		*F*_(2,158)_ = 1.061
Suicide attempts	0.81 ± 1.52	0.74 ± 1.09	0.81 ± 1.33	*F*_(2,221)_ = 0.096
Daily use of psychiatric medication	96.8%	51.7%	100%	χ^2^_(2)_ = 39.108 **
PANSS scores (for Schizophrenia diagnosis)				
Positive symptoms	8.95 ± 0.64		10.22 ± 0.73	*F*_(2,121)_ = 0.899
Negative symptoms	13.83 ± 1.65		12.15 ± 1.87	*F*_(2,121)_ = 1.210
Composite	−5.63 ± 1.40		−7.73 ± 1.81	*F*_(2,121)_ = 6.420 *
General psychopathology	24.18 ± 2.01		22.00 ± 1.82	*F*_(2,121)_ = 1.966
HDRS scores (for Major Depressive Disorder diagnosis)	9.25 ± 0.18		8.02 ± 1.05	*F*_(2,121)_ = 0.688

DD: Dual diagnosis; SUD: Substance use disorder; SMI: Severe mental illness; PANSS: Positive and negative syndrome scale; DAST-20: Drug abuse screening test; HDRS: Hamilton depression rating scale; ^a^ Percentages will not equal 100 as each participant may present more than one medical comorbidity; * *p* < 0.05; ** *p* < 0.01.

**Table 3:** Primary scale scores in the coping strategies inventory (CSI) for the three groups, with means, standard deviation, and MANCOVA results.

Coping Strategies Inventory	DD (*N* = 80)	SUD (*N* = 80)	SMI (*N* = 63)	*F*_(2,121)_	*η_p_* ^2^	Bonferroni Post-Hoc Analyses
Problem Solving	12.99 ± 0.59	12.98 ± 0.58	13.98 ± 0.58	0.23	0.002	
Self-criticism	13.33 ± 0.55	14.28 ± 0.55	10.46 ± 0.56	10.75 ***	0.089	DD, SUD > SMI
Express Emotions	10.80 ± 0.63	10.25 ± 0.63	11.09 ± 0.73	0.40	0.004	
Wishful Thinking	15.84 ± 0.47	15.51 ± 0.47	15.35 ± 0.58	0.24	0.002	
Social Support	11.07 ± 0.61	11.29 ± 0.60	11.25 ± 0.69	0.23	0.000	
Cognitive Restructuring	10.39 ± 0.57	10.09 ± 0.58	11.37 ± 0.66	1.20	0.010	
Problem Avoidance	6.68 ± 0.55	6.68 ± 0.56	10.36 ± 0.63	14.12 ***	0.114	DD, SUD < SMI
Social Withdrawal	11.68 ± 0.56	10.55 ± 0.57	11.68 ± 0.64	0.88	0.008	

DD: Dual diagnosis; SUD: Substance use disorder; SMI: Severe mental illness. *** *p* < 0.001.

**Table 4:** Follow-up descriptive data for the three groups at 3, 6, and 12 months, with means, standard deviation, or percentages.

Follow-Up Data	DD (*N* = 80)			SUD (*N* = 80)	SMI (*N* = 63)			Inter-Group Statistical Analyses (ANOVA or Chi-Square)
**3 months**		**DD with Schizophrenia (*N* = 37)**	**DD with Depression (*N* = 43)**			**SMI with Schizophrenia (*N* = 32)**	**SMI with Depression (*N* = 31)**	
Relapses	28.8%	27%	30.2%	21.3%	11.1%	6.3%	16.1%	χ^2^_(2)_ = 6.71 *
Patients’ treatment situation								χ^2^_(2)_ = 5.22
In treatment	98.8%	97.3%	100%	97.3%	100%	100%	100%	
Drop-out from treatment	1.3%	2.7%	0%	1.4%	0%	0%	0%	
Discharged from treatment	0%	0%	0%	1.4%	0%	0%	0%	
Dismissed from treatment	0%	0%	0%	0%	0%	0%	0%	
Suicide attempts	5.1%	8.1%	2.3%	2.7%	0%	0%	0%	χ^2^_(2)_ = 3.81 *
Number of medical consultations ^1^	7.6 ± 0.88	5.44 ± 1.22	9.42 ± 1.88	1.5 ± 0.51	4.3 ± 0.39	3.56 ± 0.48	4.90 ± 0.60	*F*_(2,221)_ = 22.44 ***
**6 months**								
Relapses	33.8%	32.4%	34.9%	28.8%	14.3%	9.4%	19.4%	χ^2^_(2)_ = 9.70 **
Patients’ treatment situation								χ^2^_(2)_ = 8.22 *
In treatment	91.3%	89.2%	93.3%	73.6%	98%	95%	100%	
Drop-out from treatment	5%	8.1%	4.7%	8.3%	2%	3.1%	0%	
Discharged from treatment	2.3%	0%	2%	9.7%	0%	0%	0%	
Dismissed from treatment	1.4%	2.7%	0%	4.2%	0%	1.9%	0%	
Suicide attempts	5.1%	8.1%	2.3%	6.3%	1.6%	3.1%	0%	χ^2^_(2)_ = 3.20 *
Number of medical consultations ^1^	15.71 ± 2.12	12.46 ± 2.96	18.55 ± 3.01	2.5 ± 0.74	8.4 ± 0.77	7.06 ± 0.92	9.74 ± 1.21	*F*_(2,221)_ = 23.63 ***
12 months								
Relapses	48.8%	43.2%	53.5%	38.8%	23.8%	21.9%	25.8%	χ^2^_(2)_ = 12.43 **
Patients’ treatment situation								χ^2^_(2)_ = 10.53 **
In treatment	87.2%	80%	93%	41.4%	80.5%	89.6%	90.4%	
Drop-out from treatment	9%	14.3%	4.7%	21.4%	7.1%	6.4%	3.2%	
Discharged from treatment	2.4%	2.9%	2.3%	33%	8.3%	3%	3.2%	
Dismissed from treatment	1.4%	2.8%	0%	4.2%	4.1%	1%	3.2%	
Suicide attempts	21.8%	19.5%	23.7%	5%	7.25%	9.4%	6.5%	χ^2^_(2)_ = 7.88 **
Number of medical consultations ^1^	28.12 ± 3.74	23.17 ± 5.60	31.92 ± 5.02	4.7 ± 0.92	15.97 ± 1.73	11.48 ± 1.20	20.45 ± 3.07	*F*_(2,221)_ = 19.894 ***

DD: Dual diagnosis; SUD: Substance use disorder; SMI: Severe mental illness. * *p* < 0.05; ** *p* < 0.01; *** *p* ≤ 0.001. ^1^ Medical consultations were registered if they were outside the patient’s treatment center facilities.
